# Association of visceral adiposity with increased intrarenal artery resistive index in HIV-1-infected patients receiving highly active antiretroviral therapy

**DOI:** 10.4103/2589-0557.68995

**Published:** 2010

**Authors:** Pierfrancesco Grima, Antonella Zizza, Marcello Guido, Paolo Tundo, Roberto Chiavaroli

**Affiliations:** Division of Infectious Diseases, HIV Center, “S.Caterina Novella” Hospital, Galatina, Italy; 1 Institute of Clinical Physiology, National Research Council, Lecce, Italy; 2 Laboratory of Hygiene, Department of Biological and Environmental Sciences and Technologies, Di.S.Te.B.A., Faculty of Sciences, University of Salento, Lecce, Italy

**Keywords:** HIV infection, intrarenal artery resistive index, kidney function, visceral obesity

## Abstract

**Purpose:**

The aim of our study was to evaluate whether perirenal fat thickness (PRFT), a parameter of central obesity, is related to kidney function and intrarenal artery resistive index (IARI) in human immunodeficiency virus (HIV)-1-infected patients.

**Materials and Methods:**

We enrolled 102 consecutive HIV-1-infected patients receiving highly active antiretroviral therapy for more than 12 months in a prospective cohort study. Echographically, the PRFT and IARI were measured and the serum metabolic parameters were evaluated. PRFT and IARI were measured using a 3.75 MHz convex linear probe.

**Results:**

The mean of PRFT and IARI in HIV-1-infected patients with visceral obesity was considerably higher than that in patients without it (*P* <0.001 and <0.01, respectively). Using the average IARI as the dependent variable, age (odds ratio, 1.07; 95% confidence interval [CI], 1.01–1.14; *P* < 0.5) and PRFT (odds ratio, 1.28; 95% CI, 1.08P–1.51; *P*<0.01) were independent factors associated with IARI.

**Conclusion:**

Our data indicate that ultrasonographic assessment of PRFT may have a potential to be a marker of increased endothelial damage with specific involvement of the renal vascular district in HIV-1-infected patients.

## INTRODUCTION

The introduction of highly active antiretroviral therapy (HAART) has dramatically changed the prognosis of human immunodeficiency virus (HIV) infection, with a significant decline in morbidity and mortality.[1] However, prolonged treatment with combination regimens can be difficult to sustain because of problems with compliance and toxic effects. Treatment with antiretroviral agents has uncovered a syndrome of abnormal fat redistribution, impaired glucose metabolism, insulin resistance and dyslipidemia, collectively termed lipodystrophy syndrome,[2–4] and predisposes to an increased risk of cardiovascular disease.[5,6] Visceral obesity, more than overall obesity, plays a key role in the development of cardiovascular disease.[7] Thus, estimating visceral fat distribution is important to identify subjects with a high risk for cardiovascular disease. It is known that there is a good correlation between intraabdominal fat distribution and ultrasonographic measurement of fat thickness and between perirenal fat thickness (PRFT) and central obesity.[8–12]

In addition, individuals with visceral obesity are at increased risk for progressive loss of renal function.[13] Chen *et al*.[14] demonstrated that increased waist circumference significantly correlated with microalbuminuria and glomerular filtration rate (GFR) decline, suggesting that obesity may be an independent risk for chronic kidney disease.

It is important to determine which group of HIV-1-infected patients is at an increased risk for impairment of renal function in consideration of the potential nephrotoxic action of antiretroviral drugs such as Tenofovir. The identification of predictive factors for the development of renal complication would be very useful because preventive interventions and optimized treatment could be undertaken for HIV-1- infected patients.

We performed a prospective cohort study to identify the role of visceral fat distribution on endothelial damage and risk. The objective of our study was to evaluate whether ultrasonographic parameters of visceral obesity could be associated with renal blood flow and renal function, playing a role as a possible surrogate marker of reduced renal function risk in HIV-1-infected patients.

## MATERIALS AND METHODS

This study was approved by the local institutional Ethics Committee and all patients approached for the study gave written consent for participation in the study.

### Study population

HIV-1-infected patients of Santa Caterina Novella’s Hospital (Galatina, Italy) were considered for this study. All patients had documented HIV-1 infection, had been receiving HAART for 12 months and were older than 18 years of age. An in-depth assessment was performed, including HIV disease history, other co-morbid conditions, medication exposure and measurement of blood pressure (determined by using a sphygmomanometer with the subjects in a sitting position at rest). Smokers were defined as individuals smoking more than 5 cigarettes/day at least during the past year (in our cohort all the patients who smoke, declared to smoke >5 cigarettes/day). Subjects were excluded from participation if they had any of the following clinical conditions: active acquired immunodeficiency syndrome-defining illness, diabetes mellitus or current use of oral hypoglycemic agents, family history of myocardial infarction (prior to age 55 years for first-degree male relatives and prior to age 65 years for first-degree female relatives), a history of coronary heart disease or stroke, uncontrolled hypertension, active drug abuse, alcohol abuse (defined as alcohol consumption >30 g/day), untreated hypothyroidism and chronic kidney disease. Patients requiring systemic chemotherapy, radiation therapy or systemic steroids were excluded. Two groups of patients were consecutively selected. The first group comprised of HIV-1-infected patients with a diagnosis of visceral obesity. The second group included HIV-1-infected patients for whom the diagnosis of visceral obesity had been excluded. Diagnoses of visceral obesity were based on an ultrasound-measured PRFT/body mass index ratio >0.22. Grima *et al*.[10] observed that this value could be considered a potential parameter to assess a visceral fat accumulation in HIV-1-infected patients.

### General metabolic assessment

CD4+ cells counts, HIV ribonucleic acid (RNA) load, total serum cholesterol level, high-density lipoprotein (HDL) cholesterol level, triglycerides and creatinine levels were evaluated at baseline after a 12-h overnight fast. Creatinine-based GFR was determined by the four-variable Modification of Diet in Renal Disease equation.[15]

#### Ultrasonographic measurement of visceral fat and intrarenal artery resistive index (IARI)

**Figure 1:**
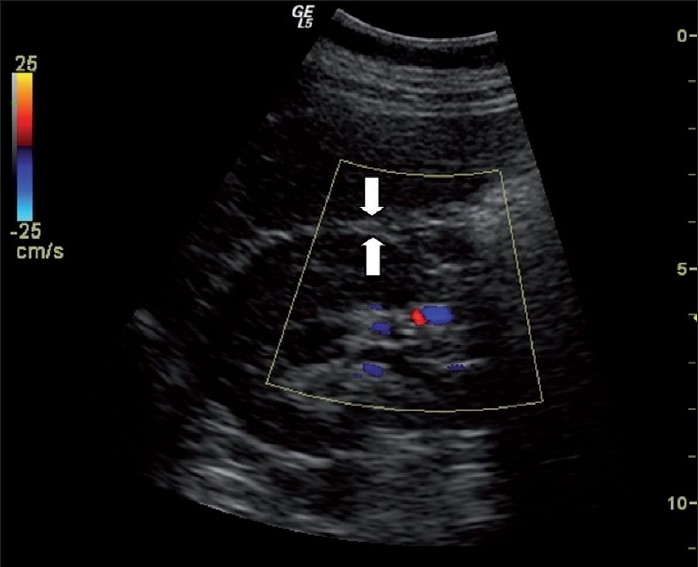
Assessment of perirenal fat diameter. Longitudinal scan (3.75 mHz) along the right midclavicular line, from the border of the right liver lobe to the border of the inferior pole of the right kidney. Arrows, limits of perirenal fat thickness

A Logiq 5 ultrasound scanner (General Electrics Medical Systems, CT, USA) equipped with a 3.75 MHz convex probe was employed. The visceral fat thickness was determined with a 3.75-MHz convex transducer at a specific referee point as PRFT (longitudinal scan along the right mid clavicular line, from the border of the right liver lobe to the border of the inferior pole of the right kidney [Figure 1]), with the patient in the supine position. IARI was measured with the patient lying supine, using an ultrasound frequency of 3.75 mHz. In each kidney, intrarenal Doppler spectra were obtained atthree representative locations from the interlobar arteriesalong the border of the medullary pyramids. The resistive index(RI) was calculated according to the following formula:

RI = 100 × (1-maximum end diastolic velocity/maximum systolic velocity)

**Figure 2:**
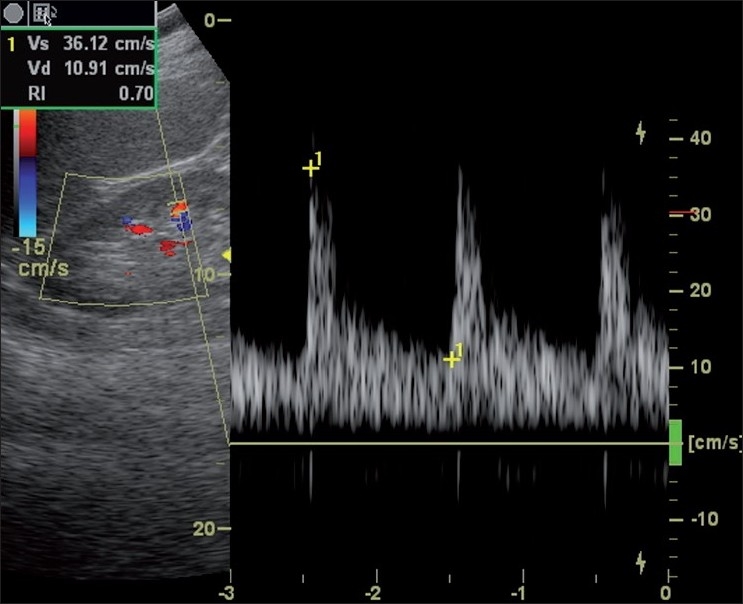
Assessment of intrarenal artery resistive index with patient lying supine using an ultrasound frequency of 3.75 mHz

Mean RI values were calculated as the averageof six RI measurements [Figure 2]. Sonographic evaluation of visceral obesity and renal artery blood flow were always performed by a single trained sonographer-blinded to the patients’ data. The reproducibility of PRFT and IARI measurement was evaluated by triple determinations in 15 subjects other than the enrolled patients. A clinically significant increase of IARI was defined as a value >0.69 according to previous observations.[16,17]

### Statistical analysis

continuous variables were reported as the mean ± standard deviation (SD) and categorical factors were reported as percentages. Demographic characteristics and metabolic variables were compared between the patient groups by analysis of variance (ANOVA) of successive measurements.

Multiple regression analysis was used to assess the independent association between the renal artery RI and PRFT, adjusting for various risk factors. The most discriminant cut-offs were calculated by receiver operating characteristic (ROC) curves. Statistical calculations were performed with MedCalc software, version 9.6.0.0. A *P*-value < 0.05 was considered to be statistically significant.

## RESULTS

### Study population

One hundred and two patients were recruited during 2009. A total of 53 patients (35 men and 18 women) were in the visceral obesity group and 49 (33 men and 16 women) were in the control group. All subjects were of Caucasian race. Table 1 shows the patient demographic and clinical characteristics.

**Table 1:** Demographic and clinical characteristics of HIV-1 infected patients receiving highly active antiretroviral therapy

Characteristic	A (n=102)	B (n=49)	C (n=53)	*P* value
Age (years)	42 ± 8.6	41.4 ± 7.	44.2 ± 8.2	NS
Sex (M/F)	78/24	39/10	39/14	NS
BMI (kg/m2)	23.51 ± 2.9	24.4 ± 1.8	23.4 ± 2.2	NS
PRFT/BMI	0.2 ± 0.09	0.31 ± 0.01	0.13 ± 0.04	<0.0001
Current smoker (%)	36.3	35.2	33.3	NS
HIV exposure				
Homosexual	48 (47.5%)	23 (46.9%)	25 (47.1%)	NS
Heterosexual	39 (38.2%)	18 (36.7%)	21 (39.6)	NS
IDU	25 (24.5%)	12 (24.4%)	13 (24.5%)	NS
HCV coinfection (%)	20.5	20	20.8	NS
CD4 (cells/μl)	535 ± 230.2	569.8 ± 263	502.8 ± 191	NS
HIVRNA load (mean log10 copies)	2.06 ± 1.2	2.1 ± 1.01	1.9 ± 1.4	NS
Duration of HAART (months)	76.8 ± 45.7	85 ± 39.9	69.8 ± 49.8	NS
Total cholesterol (mg/dl)	188.4 ± 41.8	192.5 ± 47.5	184.7 ± 35.9	NS
HDL cholesterol (mg/dl)	51.3 ± 15.8	51.6 ± 15.3	51.2 ± 16.6	NS
Triglycerides (mg/dl)	166.3 ± 95.7	176.9 ± 85.7	156.4 ± 104	NS
Creatinine clearance (ml/min/1.73m^2^)	91.9 ± 23.7	84.5 ± 18.6	98.5 ± 26	<0.01
2 NRTI + 1 NNRTI	69 (67.6%)	33 (67.3%)	36 (67.9%)	NS
2 NRTI + 1 PI	33 (32%)	16 (32.6%)	17 (32%)	NS

A-all HIV+ patients; B-HIV+ patients with visceral obesity; C-HIV+ patients without visceral obesity; BMI, body mass index; PRFT-peri-renal fat thickness; IDU-intravenous drug users; HAART-highly active antiretroviral therapy; HDL-high density lipoprotein; LDL-low density lipoprotein; NRTInucleoside reverse transcriptase; NNRTI-non nucleoside reverse transcriptase; PI-protease inhibitor; NS-not significant. All data are expressed as mean±standard deviation. A *P* value of <0.05 was considered to be statistically significant.

There were no differences between the two groups with regard to age, gender ratio, smoking status, risk factors, metabolic parameters, CD4 cells count, viral load and The triglycerides plasmatic levels and HAART duration were higher in patients with visceral obesity, although without statistical significance. Patients with diagnosis of central obesity had lower levels of creatinine clearance than that in patients without clearance (84.5 ± 18.6 vs. 98.5 ± 26 ml/min/1.73m^2^; *P* < 0.01). All HIV-1-infected patients had a backbone of Tenofovir/Emtricitabine. Protease inhibitors (PI) had been prescribed in 33 (32.3%) patients (16 with visceral obesity and 17 without obesity), without statistically significant differences between the two groups. All patients exposed to PIs received a ritonavir-boosted PI. Non-nucleoside reverse-trascriptase inhibitor had been prescribed in 69 (67.6%) patients (33 with visceral obesity and 36 without obesity).

The sonographic assessment of PRFT and IARI provided a good reproducibility, with an intraobserver variability of 6.2% and 7.4%, respectively.

At baseline, the mean of PRFT and IARI in HIV-1-infected patients with visceral obesity was considerably higher (8.1 ± 2.6 vs. 3.2 ± 0.9 mm; *P* < 0.0001, and 0.66 ± 0.05 vs. 0.63 ± 0.04; *P* < 0.001, respectively) than that in patients without visceral obesity [Table 2].

**Table 2:** Ultrasonographic PRFT and IARI measurement values in HIV-1 infected patients receiving highly active antiretroviral therapy

Characteristic	A (n=102)	B (n=49)	C (n=53)	*P* value
PRFT	5.1 ± 3	8.1 ± 2.6	3.2 ± 0.9	<0.0001*
Renal artery RI	0.64 ± 0.05	0.66 ± 0.05	0.63 ± 0.04	<0.001**

A-all HIV+ patients; B-HIV+ patients with visceral obesity; C-HIV+ patients without visceral obesity; PRFT-perirenal fat thickness; IARI-intrarenal artery resistive index. All data are expressed as mean ± standard deviation.

* B versus C;

** B versus C.

A *P* value of <0.05 was considered to be statistically significant.

**Figure 3:**
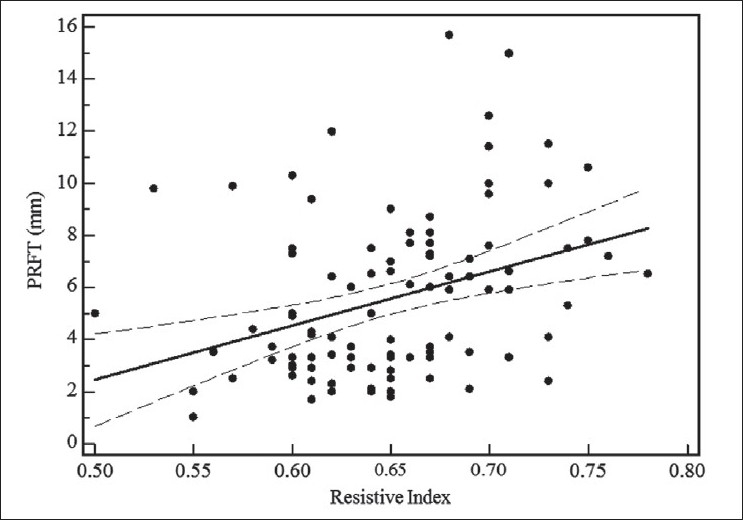
Linear regression curve of relation between echographicmeasured perirenal fat thickness and intrarenal artery resistive index in 102 human immunodeficiency virus-1-infected patients. Dotted line, 95% confidence interval; PRFT, perirenal fat thickness; RI, resistive index

**Figure 4:**
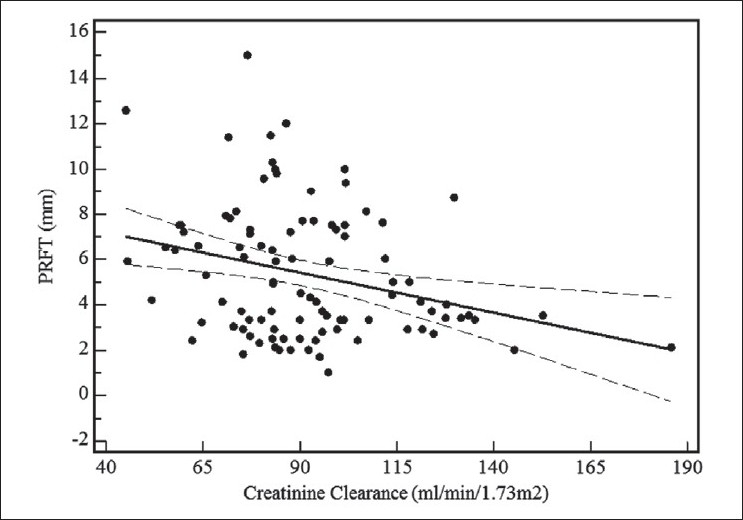
Linear regression curve of relation between echographicmeasured perirenal fat thickness (PRFT) and creatinine clearance in 102 human immunodeficiency virus-1-infected patients. Dotted line, 95% confidence interval; PRFT, perirenal fat thickness

For all 102 HIV-1-infected patients, ultrasound-measured PRFT correlated well with IARI (r +0.35; *P* < 0.001) and with creatinine clearance (r -0.29; *P* < 0.01) [Figures 3 and 4].

**Figure 5:**
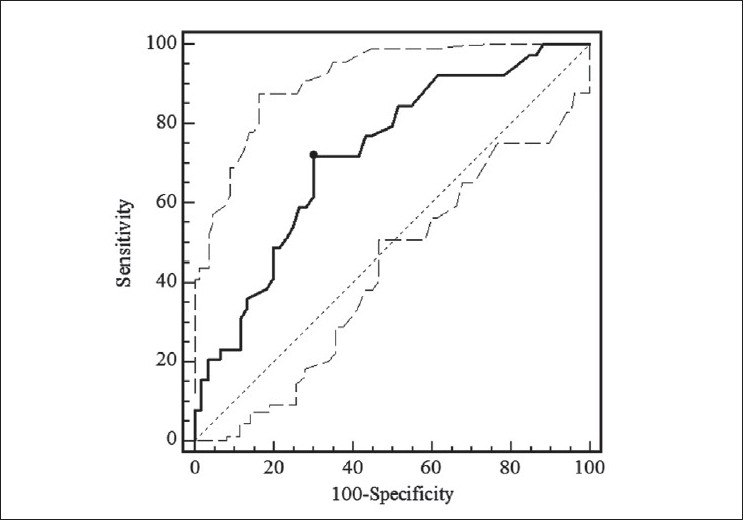
Receiver operating characteristics curves of perirenal fat thickness (PRFT) considering intrarenal artery resistive index >0.69 as status variable. The area under the curve was 0.72 (95% confidence interval, 0.62– 0.80; *P* < 0.001)

ROC indicated that the most discriminant PRFT value for predicting an IARI >0.69 was 5 mm (sensitivity 71.7%, specificity 70%, AUC 0.72, *P* < 0.001) [Figure 5]. Using the average IARI as the dependent variable, age (odds ratio, 1.07; 95% CI, 1.01-1.14; *P* < 0.05) and PRFT (odds ratio, 1.28; 95% CI, 1.08-1.51; *P* < 0.01) were independent factors associated to it.

## DISCUSSION

The introduction of HAART has induced the development of short- and/or long-term side-effects. Several reports have demonstrated that HIV-1-infected patients could have an abnormality of kidney function.[18’^20^] HAART has altered the spectrum of renal disease: the incidence of HIV-associated nephropathy has declined whereas the burden of chronic renal disease associated with diabetes, hypertension and antiretroviral therapy-induced nephrotoxicity has increased.[21,22] Moreover, many studies have shown increased visceral fat accumulation to be an independent risk factor for cardiovascular diseases, such as coronary artery disease, stroke and hypertension.[20,22] These clinical findings are also observed in kidney diseases,[23–25] signifying a potential synergistic relation with cardiovascular outcome.

Obesity has been associated with secretion of inflammatory mediators and activation of inflammation-associated signalling pathways,[26,27] with a significant role of specific mediators such as leptin, interleukin (IL)-6, tumor necrosis factor (TNF)-a and adiponectin.[28] Because many of these cytokines have been suggested to mediate renal pathophysiology,[29] it could be speculated that progressive kidney disease could also be regulated by pro-inflammatory cytokines in the context of metabolic syndrome.

From recent data, it seems that central fat distribution may be of more importance for endothelial damage than overweight by itself, confirming previous study results that demonstrated that visceral fat accumulation itself played a role as a risk factor for atherosclerosis.[11,29]

Moreover, truncal adiposity could be a significant source of TNF-α, with a direct stimulation of IL-6 followed by hepatic release of fibrinogen, C-reactive protein and plasminogen activator inhibitor-1.[25]

It was demonstrated that renal artery RI is associated with inflammation and may be a useful marker, together with albuminuria, in hypertensive patients when evaluating hypertensive renal damage.[30] Moreover, it was assessed that a significant correlation exists between RI and severity of renal disease.[31] Other studies evaluating RI in chronic renal failure have demonstrated a correlation of this index to the level of serum creatinine.[32,33]

In our study, we observed that HIV-1-infected patients with a diagnosis of visceral obesity had an increased IARI with a statistically significant reduction of creatinine clearance compared to patients without central obesity. The demonstration that PRFT in addition to age is the only independent factor associated with IARI could be considered a proof that visceral fat tissue must be considered an endocrine organ with a role in the production of atherosclerotic vascular disease.

To our knowledge, this is the first study to have demonstrated a statistically significant effect of visceral fat on renal arterial blood flow in HIV-1-infected patients, finding a reproducible tool and objective parameter for the monitoring of renal function. As a consequence, a periodic screening for visceral obesity should be considered mandatory in the HIV-1-infected patients receiving HAART.
